# Parsonage-Turner Syndrome Following Covishield (AstraZeneca ChAdOx1 nCoV-19) Vaccination: A Case Report

**DOI:** 10.7759/cureus.27867

**Published:** 2022-08-10

**Authors:** Maheshwar Lakkireddy, Sreedhar Sathu, Ravi Kumar, Karra Madhu Latha, Deepak Kumar Maley

**Affiliations:** 1 Orthopaedics, All India Institute of Medical Sciences, Bibinagar, Bibinagar, IND; 2 Orthopaedics and Trauma, All India Institute of Medical Sciences, Bibinagar, Bibinagar, IND; 3 Biochemistry, All India Institute of Medical Sciences, Bibinagar, Bibinagar, IND

**Keywords:** astra-zeneca, neuralgic amyotrophy, brachial neuritis, covid-19 vaccination, post covid vaccination parsonage-turner syndrome

## Abstract

Parsonage-Turner Syndrome (PTS) is a rare neurological disorder involving brachial plexus and periscapular muscles following viral infection, surgery, and vaccination. We hereby describe the first case of PTS from India following Covishield (AstraZeneca ChAdOx1 nCoV-19) COVID-19 vaccination.

A 21-year-old healthy male presented to us with complaints of pain and weakness in the right shoulder five weeks after Covishield vaccination on the contralateral deltoid. There was no history of injury or constitutional symptoms. On examination, hyperalgesia over the area innervated by the axillary nerve and wasting of the deltoid, supra, and infraspinatus muscles were noted. An MRI scan of the shoulder, cervical spine, and brachial plexus neurogram were normal. Decreased motor amplitude in right axillary and musculocutaneous nerve was recorded in the nerve conduction study (NCS). High titers of SARS-COV-2 IgG neutralizing antibodies were noted after a single dose of vaccination and SARS CoV-2 IgM antibodies were negative. Having been diagnosed with post-vaccination PTS, the right shoulder was splinted and an intravenous injection of 1g methylprednisolone was administered for three days followed by oral steroids for three weeks. NCS and electromyography at 10 weeks showed insignificant differences between the two sides suggesting early neurological recovery.

Currently, the patient is being followed up regularly for complete neurological recovery. PTS is a known side effect of vaccination. We report the index case of PTS following the administration of Covishield vaccination from India to aid in early diagnosis and management, further evaluation, and public health safety.

## Introduction

Parsonage-Turner syndrome (PTS), also known as brachial neuritis, neuralgic amyotrophy, is a rare neurological disorder characterized by periscapular pain, weakness of shoulder girdle muscles followed by muscle atrophy, shoulder subluxation, or pathological dislocation. It is a rare sequel of viral infection, surgery, and vaccination [1-2]. Being a rare disorder of idiopathic etiology, PTS is often misdiagnosed or undiagnosed early in its course and hence may end up in lifelong disability if it is not managed appropriately. A high index of clinical suspicion is necessary while treating such cases. COVID-19 vaccination is currently perceived as the best solution for public health safety and the only hope for a revival of economies across the globe. To date, this is the largest vaccination drive conducted in this country in a relatively short period of time, making it imperative to report any adverse event following immunization against COVID-19 in order to contribute to the public health safety database. Having noted brachial neuritis as one of the rare adverse effects following immunization, we report a case of PTS following administration of the COVID-19 AstraZeneca ChAdOx1 nCoV-19 vaccine which is marketed as Covishield in India.

## Case presentation

**Figure 1:**
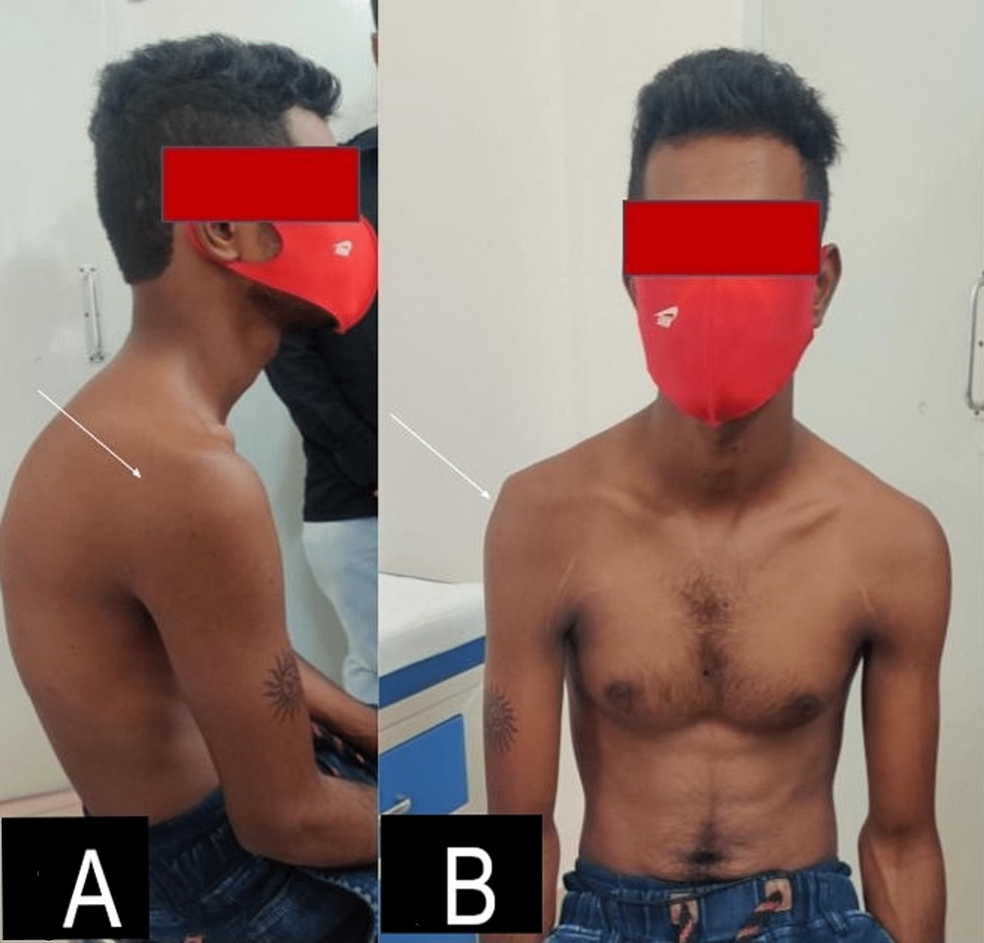
Clinical picture showing wasting of the right deltoid (white arrows) from the side (A) and from the front (B) at five weeks post-COVID-19 vaccination respectively

A 21-year-old healthy male presented to the orthopedic outpatient department with complaints of pain and weakness in the right shoulder for four weeks. He was apparently asymptomatic five weeks ago when he received the Covishield vaccine in the left deltoid. One week after the vaccination, he developed insidious onset pain and a burning sensation in the right shoulder. Symptoms have progressed from mild to severe intensity in the next one week. He was treated with analgesics before he presented to us and had no relief. Three weeks after vaccination, he experienced difficulties when using the right shoulder joint. There was no history of injury to the neck or shoulder nor fever or any constitutional symptoms following or preceding the vaccination as a possible alternative explanation of the weakness. Clinical examination at five weeks following vaccination (at presentation) has revealed significant wasting of deltoid with mild wasting of supraspinatus and infraspinatus muscle bulk with hyperalgesia over the area innervated by the axillary nerve (Figure 1A-1B).

**Figure 2:**
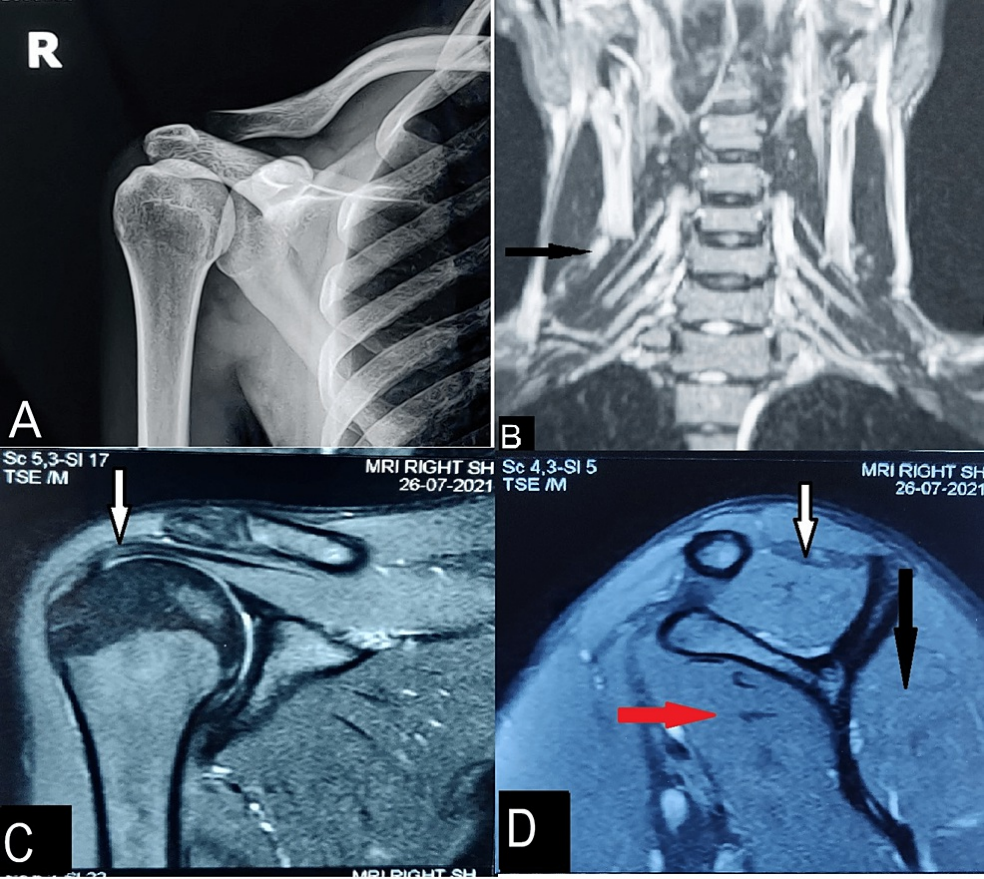
Radiological images at five weeks post-vaccination Normal X-ray anteroposterior view of the affected shoulder (A). Neurogram of upper brachial plexus showing intact nerves (B). T1 coronal MRI image of affected shoulder showing intact tendon (C). T1 sagittal MRI image showing normal supraspinatus, infraspinatus, and subscapularis muscle bulk represented with white, black, and red arrows respectively (D).

The active range of motion of the right shoulder joint was completely lost. There was no evidence of winging of the scapula. The ipsilateral elbow, wrist, and joints in the hand were apparently normal. He was evaluated (outside our hospital) with a radiograph of the right shoulder, magnetic resonance imaging (MRI) of the cervical spine, brachial plexus neurogram, and MRI of the shoulder joint. No abnormality was observed in all the radiological investigations (Figure 2A-2D).

A nerve conduction study performed on the right upper limb showed decreased compound motor action potential in the axillary and musculocutaneous nerve, however, no clinically evident weakness in muscles supplied by the musculocutaneous nerve was noted (Table 1). 

**Table 1:** Nerve conduction study of the right axillary, musculocutaneous, median, and ulnar nerves at five weeks post-vaccination N: nerve

Site		Latency 1 (mS)	Duration (mS)	Amplitude	NCV (m/S)
Median N	Wrist	3.13	11.88	9.8 mV	
	Elbow	7.08	12.29	9.3 mV	60.61
Ulnar N	Wrist	1.98	10.94	12.6 mV	
	Elbow	6.15	11.35	12.3 mV	59.95
Axillary N	Erb’s Point	10.83	8.65	2.6 mV	
Musculocutaneous N	Erb’s Point	8.54	8.23	3.1 mV	

**Figure 3:**
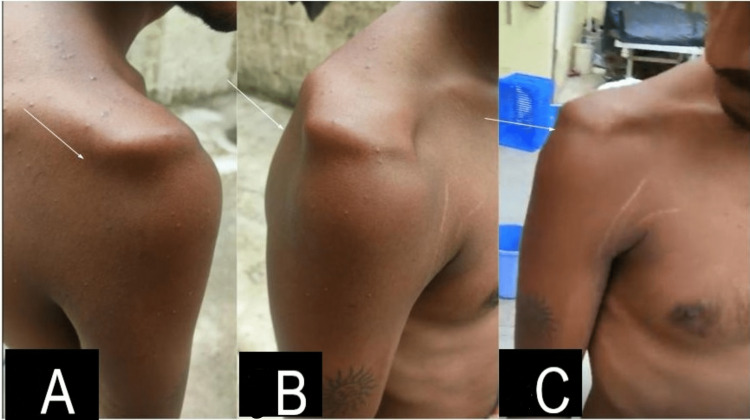
Clinical picture showing papular eruptions over the right shoulder and gross wasting of the deltoid and infraspinatus muscle (white arrow) from the back (A), from the side (B), and from the front (C) respectively at seven weeks post-vaccination.

Under the supervision of a neuro physician, an intravenous injection of 1 g of methylprednisolone was administered for three days followed by 12 mg of deflazacort twice daily for two weeks and once daily for one week. Pregabalin 75 mg twice daily was added as an adjunct along with an arm sling pouch splint. Owing to peer pressure, the patient visited a quack and received treatment in the form of massaging and bandaging. Meanwhile, he developed gross wasting of supra and infraspinatus muscles along with deltoid and increasing inferior subluxation of the shoulder joint. He developed multiple papular eruptions (non-dermatomal in distribution) over the areas of herbal medicine application (Figure 3A-3C).

As he came back to follow up, he was reviewed with the dermatologist to rule out herpes infection. Tzanck smear and histopathology examination of the lesions showed plenty of epithelial cells and did not yield any organisms. Haemogram, erythrocyte sedimentation rate (ESR), and C-reactive protein (CRP) were normal. A provisional diagnosis of acne vulgaris of the torso secondary to herbal medication/steroid usage was made. Lesions subsided after topical usage of benzoyl peroxide 2.5% w/w and clindamycin ointment. Nine weeks following the first dose of the Covishield vaccine, he developed significantly high titers of SARS -COV-2 IgG neutralizing antibodies (Result: >20 index units, non-reactive <1.0, reactive >=1) and SARS CoV-2 IgM antibodies were negative. Nerve conduction study and electromyography at 10 weeks showed insignificant differences between the two sides suggesting early neurological recovery to be followed by the gradual recovery of muscle bulk (Table 2).

**Table 2:** Nerve conduction study showing the insignificant difference between the two sides suggesting early neurological recovery 10 weeks post-vaccination mV: millivolts, mS: milliseconds, N: nerve, APB: abductor pollicis brevis, ADM: abductor digiti minimi, Erb's P: Erb's Point, SSP: supraspinatus muscle, ISP: infraspinatus muscle.

Site				Latency (mS)	Amplitude (mV)
Right	Musculocutaneous N	Erb’s P - Biceps		4.3	16.4
	Axillary N	Erb’s P - Deltoid		4.2	11.9
	Suprascapular N		Erb’s point - SSP	2.3	6.1
			Erb’s point - ISP	4.8	1.8
	Median N		Wrist - APB	3.1	14.7
			Elbow - Wrist	7.0	13.0
	Ulnar N		Wrist - ADM	2.2	10.5
			B. Elbow - Wrist	5.8	9.6
			A. Elbow - B. Elbow	7.2	8.7
Left	Musculocutaneous N	Erb’s P - Biceps		4.1	15.6
	Axillary N	Erb’s P - Deltoid		4.2	16.6
	Suprascapular N		Erb’s P - SSP	4.4	6.6
			Erb’s P - ISP	4.4	1.7
	Median N		Wrist - APB	2.9	10.0
			Elbow - Wrist	6.8	9.8
	Ulnar N		Wrist - ADM	2.0	10.3
			B. Elbow - Wrist	5.9	10.0
			A. Elbow - B. Elbow	7.6	9.6

Currently, the patient is being followed up regularly with advice to support the affected shoulder and perform passive range of motion exercises to avoid stiffness and deformity while complete neuromuscular improvement ensues over time.

## Discussion

PTS is a rare neurological disorder of idiopathic etiology. Most commonly PTS presents as sudden onset of shoulder and periscapular pain followed by weakness and muscle atrophy. One in 1000 is the overall estimated incidence of PTS [1,3]. In the USA, 18 cases of brachial neuritis have been reported following 350 million doses of vaccination in the 2018-2020 period [4]. The annual incidence of acute brachial neuritis in the UK is three cases per 100 000 [5].

Several risk factors have been proposed in the development of PTS and the commonest is the recent viral illness (25%) followed by vaccination (15%) [6]. One PTS case and one case of abducent nerve palsy were reported after the COVID-19 vaccination (Pfizer - BioNTech) from the USA [7-10]. Few neurological phenomena have been documented as potential side effects of the Covishield (Astra Zeneca: ChAdOx1 nCoV-19) vaccine [9-11] and it is crucial to report such adverse drug effects arising from it to aid in global vaccination effort and to stop the spread of misinformation.

In this index case, we report the first case of PTS following the Covishield vaccination from India wherein a 21-year-old gentleman presented to us with right shoulder pain and weakness following the administration of the Covishield vaccine on his contralateral deltoid. Clinical signs and symptoms with sensory-motor involvement, unilaterality of affection, nerve conduction abnormality, high titers of neutralizing antibodies after a single dose of Covishield vaccination were in favor of post-vaccination PTS, akin to literature. Considering the sequence of events favoring the diagnosis and after ruling out other potential causes, we report this adverse event following vaccination after notifying the concerned authorities for public health safety.

Therefore, in light of the above findings, it is imperative to investigate the pathophysiology of PTS or any other neurological phenomenon associated with AstraZeneca ChAdOx1 nCoV-19 vaccination [9-11]. It is recommended that clinicians and health authorities should be watchful and vigilant to look for the adverse events following immunization to Covishield vaccination to support or refute the association. Our effort in reporting this index case would be helpful for early diagnosis and appropriate treatment of PTS following the COVID vaccination.

## Conclusions

PTS is a known side effect of vaccination. It is characterized by variable involvement of brachial plexus leading to sensorimotor insufficiency of the involved part. Early detection and appropriate treatment are of paramount importance to avoid lifelong disability. Having diagnosed and treated the patient appropriately, we report the first case of PTS following Covishield (Astra Zeneca ChAdOx1 nCoV-19) vaccination from India to aid in early diagnosis and management, further evaluation, and public health safety.
